# Complete Genome Sequence of *Dietzia* sp. Strain WMMA184, a Marine Coral-Associated Bacterium

**DOI:** 10.1128/genomeA.01582-17

**Published:** 2018-02-01

**Authors:** Doug R. Braun, Marc G. Chevrette, Deepa Acharya, Cameron R. Currie, Scott R. Rajski, Kim B. Ritchie, Tim S. Bugni

**Affiliations:** a Pharmaceutical Sciences Division, University of Wisconsin—Madison, Madison, Wisconsin, USA; b Department of Bacteriology, University of Wisconsin—Madison, Madison, Wisconsin, USA; c Laboratory of Genetics, University of Wisconsin—Madison, Madison, Wisconsin, USA; d The University of South Carolina—Beaufort, Beaufort, South Carolina, USA

## Abstract

*Dietzia* sp. strain WMMA184 was isolated from the marine coral *Montastraea faveolata* as part of ongoing drug discovery efforts. Analysis of the 4.16-Mb genome provides information regarding interspecies interactions as it pertains to the regulation of secondary metabolism and natural product biosynthesis potential.

## GENOME ANNOUNCEMENT

Over the last decade, initiatives to identify and develop new chemotypes as tools in the fight against drug resistance have focused, in large part, on devising ways to activate otherwise dormant or “cryptic” biosynthetic gene clusters (BGCs) within microbial organisms (1–3). One means by which this has been accomplished involves the coculturing of two or more microbes within the same vessel; such fermentations often trigger the production of natural products that would otherwise not be produced by virtue of their BGC dormancy (4, 5). It is now clear, as reflected both in the lab and in naturally occurring microbiome systems (6), that microbial cross-communications (both competitive and collaborative in nature) enable the production of small-molecule secondary metabolites that are otherwise unattainable; BGCs for such compounds in the absence of other microbial stimuli remain silent and nonproductive. Coculturing approaches to new chemotypes dictate the importance of genomic data for cocultured organisms; the diversities attainable by such new chemotypes/structures stem, in large part, from the diversity of cocultured organisms (7, 8). In light of these considerations, it is noteworthy that mycolic acid-producing bacteria inclusive of, but not limited to, the genera *Nocardia*, *Mycobacterium*, and *Dietzia* are known to effectively activate actinorhodin and undecylprodigiosin BGCs in *Streptomyces lividans* (9).

To date, there have been only 14 *Dietzia* assemblies deposited in GenBank that are representative of organisms isolated from widely varied environments (10–20); some of these represent significant human pathogens or candidate pathogens (10, 13, 15–18). Marine-derived *Dietzia* representatives are well-known, although only two, *Dietzia alimentaria* 72^T^ from the Korean seafood *jeotgal* (19), and *Dietzia* sp. strain 111N12-1 from seawater samples from the South China Sea (20), have been rigorously sequenced and deposited to GenBank thus far. This report, as part of our coculture initiatives to identify new antimicrobial chemotypes, signals the GenBank deposition of the third marine-derived *Dietzia* genome sequence.

*Dietzia* sp. strain WMMA184 was isolated in 2011 from coral mucus of *Montastraea faveolata* collected off the coast of the Florida Keys. WMMA184 was isolated from a plate prepared using M1 medium (21) supplemented with 50% artificial seawater (ASW).

The complete genome of *Dietzia* sp. WMMA184 was sequenced at the Duke Center for Genomic and Computational Biology (GCB) using PacBio RS II (Pacific Biosciences) technology. Reads were assembled using the HGAP assembler (22) into six contigs. Open reading frames were predicted by Prodigal (23) and annotated using the Rapid Annotation using Subsystems Technology (RAST) software (24). The genome was found to be 4.16 Mb in length, with a GC content of 69.9%. The biosynthetic potential of the organism was assessed using antiSMASH 4.0 (25) and PRediction Informatics for Secondary Metabolomes (PRISM) (26). Out of 48 putative gene clusters identified, there are 2 terpene clusters, one type I polyketide/saccharide hybrid cluster, and one siderophore BGC housed in the WMMA184 genome.

### Accession number(s).

The complete genome sequence of *Dietzia* sp. WMMA184 has been deposited at DDBJ/EMBL/GenBank under the project accession number NXEI00000000, which correlates to BioProject PRJNA400578.
